# High Stability and Corrosion‐Resistant Gas of Recyclable and Versatile Manganese‐Doped Lead‐Free Double Perovskite Crystals toward Novel Functional Fabric and Photoelectric Device

**DOI:** 10.1002/advs.202403352

**Published:** 2024-06-14

**Authors:** Xiaoman Zhang, Xuyi Wang, Kun Nie, Xiuqiang Duan, Ziyao Hu, Xiaodong Zhang, Lefu Mei, Luoxin Wang, Hua Wang, Xiaoxue Ma

**Affiliations:** ^1^ School of Materials Science and Engineering Hubei Key Laboratory for New Textile Materials and Applications and State Key Laboratory of New Textile Materials & Advanced Processing Technology Wuhan Textile University Wuhan 430200 P. R. China; ^2^ China Bluestar Chengrand Co. Ltd. High‐Tech Organic Fibers Key Laboratory of Sichuan Province Chengdu 610042 P. R. China; ^3^ School of Materials Science and Technology Engineering Research Center of Ministry of Education for Geological Carbon Storage and Low Carbon Utilization of Resources Beijing Key Laboratory of Materials Utilization of Nonmetallic Minerals and Solid Wastes National Laboratory of Mineral Materials China University of Geosciences (Beijing) Beijing 100083 P. R. China

**Keywords:** high stability luminescent fibers, lead‐free double perovskites, perovskite devices, photosensitive fiber filter bags

## Abstract

Lead‐free halide perovskites possess excellent photoelectric properties, making them widely used in the photoelectric fields. Herein, lead‐free double perovskite crystals (PCs) doped with manganese (Cs_2_NaInCl_6_:Mn^2+^) are successfully prepared by the more energy‐efficient crystallization method. The crystals emit bright orange‐red light under the ultraviolet (UV) lamp, showing unique optical properties. They have the highest photoluminescence quantum yield of 42.91%. The white light‐emitting diodes (LEDs) are fabricated using these perovskite crystals, which show a color rendering index of 92 and external quantum efficiency (EQE) as high as 16.3%. Furtherly, perovskite‐modified fiber paper made of aramid chopped fibers (ACFs) and polyphenylene sulfide (PPS) exhibited fluorescent properties under different conditions. This paper combines fiber composite technology with PPS fiber filter bags, which are widely used in environmental protection, for the first time and demonstrates functional fiber filter bags with fluorescent characteristics. This filter bag provides an idea for the automatic detection of industrial filtration. Meanwhile, after being exposed to industrial waste gas for 60 h, the filter bag can maintain superior fluorescence performance. In this study, lead‐free double perovskites are synthesized using an efficient method for preparing high‐performance LEDs and high‐stability fluorescent fibers. Concurrently, the application of perovskites in environmental protection is expanded.

## Graphical abstract

Manganese‐doped halide perovskites with high photoluminescent quantum yield can be used to prepare LEDs and fluorescent composite fibers with high external quantum efficiency. The fluorescent fiber filter bag is developed based on fluorescent composite fiber, which provides a new idea for detecting filter bag damage in industrial waste gas filtration systems.

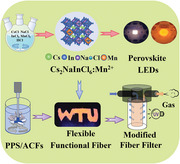

## 1 Introduction

Lead halide perovskite APbX_3_ (A = Cs^+^, MA^+^, FA^+^; X = Cl^−^, Br^−^, I^−^) exhibits outstanding properties in optical performance, including high carrier mobility, adjustable spectral absorption range, and near‐unity photoluminescence quantum yield (PLQY).^[^ ^1^, ^2^, ^3^ ^]^ These properties make it widely used in optoelectronics, such as fluorescence detection and solar cell applications.^[^ ^4^, ^5^ ^]^ However, toxicity and instability limit the scope of its commercial application.^[^ ^6^, ^7^ ^]^ Lead‐free perovskite with the formula A_2_B^+^B^3+^X_6_ (A = Cs^+^, MA^+^, FA^+^; B^+^ = Ag^+^, K^+^, Na^+^, Li^+^; B^3+^ = Bi^3+^, Sb^3+^, In^3+^; X = Cl^−^, Br^−^, I^−^) is equipped with relatively improved thermal stability and a wide adjustable optical band gap via B sites transmutation or exotic doping and reveal the significant potential in the field of optoelectronics.^[^ ^8^, ^9^, ^10^ ^]^ As a classical structure in lead‐free double perovskite, Cs_2_NaInCl_6_ has received particular attention due to its direct bandgap structure. However, due to the parity‐forbidden transition restriction, it has a low PLQY, making it challenging to play a significant role in practical applications.^[^ ^11^, ^12^, ^23^ ^]^ Doping of Cs_2_NaInCl_6_ with other metal elements will significantly improve its fluorescence performance.^[^ ^16^, ^17^, ^32^ ^]^ Therefore, it is essential to research the change in optical properties of Cs_2_NaInCl_6_ by doping different metal elements. The doping of other elements on intrinsic semiconductors can change the photoelectric properties of semiconductors.^[^ ^14^, ^18^, ^19^, ^21^ ^]^ Due to the effective ^4^T_1_→^6^A_1_ d‐d transition from Mn^2+^ due to their long lifetime, Mn^2+^‐doped semiconductors have shown various applications, including biomedical imaging, solar cells, and temperature sensors.^[^ ^22^, ^23^, ^24^ ^]^ In Cs_2_NaInCl_6_:Mn^2+^ PCs, manganese replaces indium as a new emission center to produce a new emission peak. Cs_2_NaInCl_6_:Mn^2+^ PCs can emit an orange‐red light. Although manganese‐doped Cs2NaInCl_6_ perovskite has previously been reported, using high‐temperature and high‐pressure hydrothermal methods is not conducive to large‐scale industrial production or producing a product with a low PLQY.^[^ ^22^, ^24^, ^25^ ^]^ The study reports the Cs_2_NaInCl_6_:Mn^2+^ PCs synthesized by co‐precipitation at low temperatures and atmospheric pressure. The PLQY of Cs_2_NaInCl_6_:Mn^2+^ PCs was as high as 42.91%.

Meanwhile, LEDs based on perovskite are widely used in lighting because of their high chromaticity, wide color gamut, and low cost. We made Cs_2_NaInCl_6_:Mn^2+^ PCs into orange‐red and white LEDs based on their excellent optical performance. The color rendering index of white LEDs reached 92, and EQE was as high as 16.3%. Those indicate that they have potential in the field of LEDs. In addition, fiber paper made of ACFs and PPS was modified by perovskite and produced fluorescence under the UV lamp.^[^ ^28^, ^31^ ^]^ The flexible luminous fabric modified by Cs_2_NaInCl_6_:Mn^2+^ perovskite retains the original structure and flexibility. It can produce fluorescent luminescence and maintain its good luminous performance in high temperature, low temperature, and sunshine. This study provides new ideas for the research direction of the wearable, flexible luminous fiber. PPS fiber filter bags perform well when applied to industrial waste gas. Based on the modified functional fiber and PPS fiber filter bag, a fluorescent fiber filter bag was further designed and prepared for industrial waste gas filtration.^[^ ^33^, ^34^, ^35^ ^]^ When a specific filter bag area is damaged, a particular wavelength of UV light is irradiated, and the photosensitive detector cannot detect the fluorescence. The relevant data are transmitted to the detection system to complete the automatic detection. Meanwhile, the filter bag can still maintain good fluorescence characteristics when used for tens of hours in some industrial waste gas environments (CO_2_, SO_2_, HCl). This research provides a direction for using perovskites in environmental protection fields such as carbon neutrality. Further, it broadens the use of lead‐free perovskites in chemical engineering and flexible, functional fiber.

## 2 Result and Discussion

**Figure 1:**
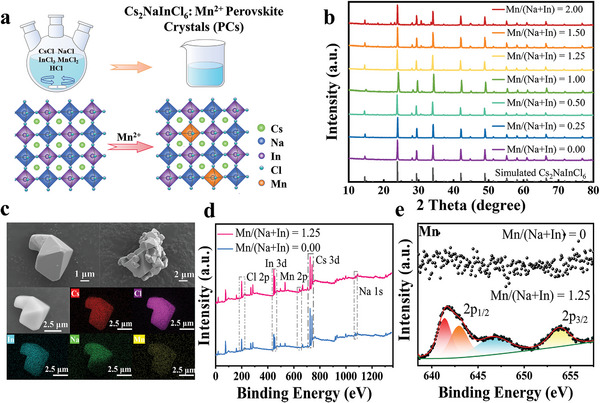
a). Schematic diagrams of the synthesis of Cs_2_NaInCl_6_:Mn^2+^ PCs and the crystal structures of Cs_2_NaInCl_6_ and Cs_2_NaInCl_6_:Mn^2+^ PCs. b) XRD patterns of the Cs_2_NaInCl_6_ with the different ratio doping of Mn^2+^. c) SEM images of Cs_2_NaInCl_6_ PCs doped with Mn^2+^ and elemental mapping images of Cs, In, Na, Cl, and Mn in Cs_2_NaInCl_6_:Mn^2+^ PCs. d) XPS spectra of Cs_2_NaInCl_6_ and Cs_2_NaInCl_6_:Mn^2+^. e) High‐resolution XPS spectra of Mn.

Cs_2_NaInCl_6_:Mn^2+^ PCs are prepared using a more energy conservation and efficient co‐precipitation method (**Figure** **1**a; details are given in the experimental section). Figure 1a shows the schematic structure of Cs_2_NaInCl_6_:Mn^2+^ PCs. The corner connected [InCl_6_]^3−^ and [NaCl_6_]^5−^ octahedron forms a 3D framework, and Cs^+^ is in the cavities of the octahedron.^[^ ^15^ ^]^ To maintain charge balance during Mn^2+^ doping, Mn^2+^ usually replaces Na^+^ and In^3+^ in the original Cs_2_NaInCl_6_:Mn^2+^ PCs and forms [MnCl_6_]^4−^ octahedron. The X‐ray diffraction (XRD) patterns of the Cs_2_NaInCl_6_ with the different ratios of Mn^2+^ showed high signal‐to‐noise ratios and possessed the same cubic phase of the Cs_2_NaInCl_6_ PCs structure. These results agreed with the simulated results of pure Cs_2_NaInCl_6_, indicating its reliable purity and great crystallization.^[^ ^10^ ^]^ Figures 1c and Figures S1a (Supporting Information) showed the scanning electron microscope (SEM) images of un‐doped Cs_2_NaInCl_6_ and the Cs_2_NaInCl_6_:Mn^2+^ (Mn/(Na+In) = 1.50). Cs_2_NaInCl_6_:Mn^2+^ PCs can be observed that most sizes in several are 5–8 µm and have an octahedral crystal morphology, which the crystal Cs_2_NaInCl_6_ of after modification is almost unchanged. Distinct octahedral crystal morphology can also be observed under SEM images of large crystals agglomerated by multiple single crystals (Figure S1a, Supporting Information). Moreover, the elemental mapping of energy dispersive spectrometer (EDS) of Cs, Na, In, Mn, and Cl in Cs_2_NaInCl_6_:Mn^2+^ and map sum spectrum of Cs_2_NaInCl_6_:Mn^2+^ indicate that the elements are uniformly distributed, which proves the reliability of the crystallization method (Figures 1c; Figure S1b, Supporting Information). To confirm the Mn^2+^ were successfully doped into Cs_2_NaInCl_6_:Mn^2+^ PCs, electron paramagnetic resonance (EPR) measurements were performed to identify the oxidation state and the local environment of the Mn^2+^ in the synthesized double perovskites. The room‐temperature (RT) EPR spectra of the Cs_2_NaInCl_6_:Mn^2+^ (Mn/(Na+In) = 1.25) displayed the characteristics of a single broad spectrum, with the g‐factor of 2.0047 and a hyperfine constant A of 8.4 mT, which probably confirms the incorporation of Mn^2+^ cations into the PCs structure (Figure S2, Supporting Information). A single broad spectrum implies the formation of an Mn^2+^ cluster due to exchange interaction between magnetically coupled Mn^2+^ pairs.^[^ ^22^, ^26^ ^]^ This single spectrum also occurs when other magnetic elements are abundant. Therefore, X‐ray photoelectron spectroscopy (XPS) was employed to examine the chemical composition of Mn^2+^‐doped PCs and pure Cs_2_NaInCl_6_ PCs (Figure 1d,e). Cs 3d, Na 1s, In 3d, and Cl 2p were consistent with Cs_2_NaInCl_6_. The Mn 2p_1/2_ and Mn 2p_3/2_ peaks were inconsistent with that of Cs_2_NaInCl_6_, proving that Cs_2_NaInCl_6_ had been doped with Mn^2+^ successfully (Figure 1e; Figure S3, Supporting Information).

**Figure 2:**
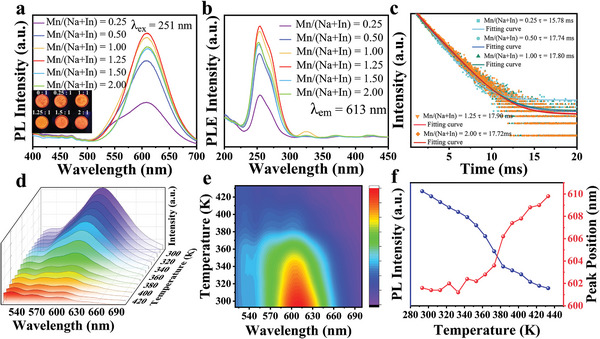
a). PL spectra of Cs_2_NaInCl_6_:Mn^2+^ (Mn/(Na+In) = 0.25, 0.50, 1.00, 1.25, 1.50, and 2.00) PCs. The Fluorescence of Cs_2_NaInCl_6_:Mn^2+^ PCs under 254 nm UV light. b) PLE spectra of Cs_2_NaInCl_6_:Mn^2+^ (Mn/(Na+In) = 0.25, 0.50, 1.00, 1.25, 1.50, and 2.00) PCs. c) TRPL decay curves of Cs_2_NaInCl_6_:Mn^2+^ (Mn/(Na+In) = 0.25, 0.50, 1.00, 1.25, and 2.00) PCs. d) Temperature‐dependent PL spectra of Cs_2_NaInCl_6_:Mn^2+^ (Mn/(Na+In) = 1.25. e) Temperature‐dependent optical behaviors of the Cs_2_NaInCl_6_:Mn^2+^ PCs. f) Integrated PL intensity and PL peak position as a function of temperature.

The optical properties of Cs_2_NaInCl_6_ and Cs_2_NaInCl_6_:Mn^2+^ PCs crystals were tested. The photoluminescence (PL) spectrum of the pure Cs_2_NaInCl_6_ shows an emission peak at 441 nm, which is excited by 318 nm UV light (Figure S4a,b, Supporting Information). This result is consistent with previous literature, whose broad emission bands and significant Stokes shifts are all characteristic of self‐trapped emission (STE).^[^ ^13^ ^]^ With the gradual increase of the ratio of Mn^2+^, the intensity of the emission peak at 441 nm gradually decreases under the 318 nm UV lamp. In comparison, a weak emission peak appears at 620 nm (Figure S8, Supporting Information). Meanwhile, under the irradiation of 251 nm ultraviolet lamp, there was no emission peak in the pure Cs_2_NaInCl_6_, but a strong emission peak appeared at 613 nm after adding Mn^2+^(Figure S4c,d, Supporting Information). Those results indicate that Mn^2+^ gradually replaces Na^+^ and In^3+^ as new emission centers. PL spectra of Cs_2_NaInCl_6_:Mn^2+^ PCs show the band of all Cs_2_NaInCl_6_:Mn^2+^ PCs excited with the 251 nm UV light, broadly covered from 520 to 710 nm and peaked at 613 nm (**Figure** **2**a). When the ratio of Mn^2+^ is less than 1.25, the PL intensity increases gradually with the increase of the ratio. The intensity is the highest at the ratio of 1.25 and decreases when the Mn^2+^ continues to increase doping. According to previous reports, this reduction in PL intensity was mainly due to the concentration quenching effect.^[^ ^30^ ^]^ The trend of PL corresponds to that of photoluminescence excitation (PLE) (Figure 2b). The synthesized Cs_2_NaInCl_6_:Mn^2+^ PCs emit a bright orange‐red light under the 254 nm deep ultraviolet (d‐UV) lamp (Figure 2a inset). The XRD patterns and PL spectra of Cs_2_NaInCl_6_:Mn^2+^ PCs prepared by different reaction temperatures and times are demonstrated (Figures S5–S7, Supporting Information). With the increase in synthesis temperature and the rise in synthesis time, the fluorescence intensity first increased and then decreased, showing reasonable regularity. The PL intensity is the highest when the synthesis temperature is 80 °C and the synthesis time is 1 h. Figure S10a,b (Supporting Information) shows the PLE and PL spectra of Cs_2_NaInCl_6_:Mn^2+^ (Mn/(Na+In) = 1.25) PCs with different monitoring and excitation wavelengths. It can be observed that regardless of the changes in wavelengths of excitation and emission, the wave profile of PL and PLE spectra remains unchanged, which proves that the luminescence characteristics of Cs_2_NaInCl_6_:Mn^2+^ PCs do not come from the lattice or surface defects of crystal.^[^ ^30^ ^]^ Figure S10c,d (Supporting Information) shows the 2D wavelength scanning spectrum and 3D wavelength scanning spectrum of Cs_2_NaInCl_6_:Mn^2+^ (Mn/(Na+In) = 1.25) PCs, which indicate the corresponding emission wavelength under a certain excitation wavelength.

To further understand the optical behaviors of the Cs_2_NaInCl_6_:Mn^2+^ PCs. Time‐resolved PL (TRPL) decay curves of Cs_2_NaInCl_6_:Mn^2+^ PCs can be fitted with the bi‐exponential Equation (1):^[^ ^26^ ^]^

(1) $$I\left(t\right)=A_{1}\;\exp\left(-t/\tau_{1}\right)+A_{2}\exp\left(-t/\tau_{2}\right)$$

The average fluorescence lifetime can be calculated using Equation (2):^[^ ^20^ ^]^

(2) $$\tau_{avg}=\;\left(A_{1}\tau_{1}^{2}+A_{2}\tau_{2}^{2}\right)/\left(A_{1}\tau_{1}+A_{2}\tau_{2}\right)$$

where 𝐴_1_ and 𝐴_2_ are the amplitudes and τ_1_ and τ_2_ represent the fast and slow decay lifetimes, respectively. It can be calculated that the average fluorescence lifetimes of Cs_2_NaInCl_6_:Mn^2+^ PCs are 15.78 ms (Mn/(Na+In) = 0.25), 17.74 ms (Mn/(Na+In) = 0.50), 17.80 ms (Mn/(Na+In) = 1.00) 17.90 ms (Mn/(Na+In) = 1.25) and 17.72 ms (Mn/(Na+In) = 2.00) (Figure 2c). At the same time, combined with the PL spectra, the emission intensity of the Cs_2_NaInCl_6_:Mn^2+^ is positively related to its lifetime (Figure 2a,c). The maximum fluorescence lifetime of 17.90 ms is the longest reported in Mn^2+^‐doped II‐VI semiconductors or perovskite materials. According to previous reports, this reduction in PL and PL Lifetime was mainly due to the concentration quenching effect.^[^ ^9^, ^15^, ^27^ ^]^ Such a long lifetime entirely excludes the possibility of STE emission, which generally has a lifetime on a scale of 10^−6^ s.^[^ ^12^ ^]^ Thus, it is strongly suggested that the emission originates from the spin‐forbidden ^4^T_1_→^6^A_1_ d‐d transition of excited.^[^ ^29^ ^]^ The exchange coupling of the Mn^2+^ dopant and its surrounding self‐trapped state undergoes an energy transfer process, which excites the manganese dopant. The orange‐red light emission can be attributed to the ^4^T_1_ → ^6^A_1_ transitions from the octahedral coordination Mn^2+^ (Figure S9, Supporting Information).^[^ ^13^ ^]^ Furthermore, the lifetime is almost an order of magnitude longer than that in other hosts, such as II–VI semiconductors, indicating the relatively strong forbidding of the electronics transition of Mn^2+^ in the investigated Cs_2_NaInCl_6_:Mn^2+^ PCs. The much longer lifetime of Mn^2+^ emission in Mn^2+^‐doped PCs may be due to the different location and crystal field environments of Mn^2+^ dopants. The II‐VI semiconductor lattices have tetrahedral cation symmetry. However, Mn^2+^‐doped perovskite crystals have pseudo‐octahedral symmetry. The inversion symmetry at Mn^2+^ sites in Mn^2+^‐doped perovskite lattices will make a ^4^T_1_→^6^A_1_ transition.^[^ ^12^ ^]^ The transition is parity‐forbidden and spin‐forbidden, resulting in a longer PL lifetime of Mn^2+^. The highest PLQY in all the products synthesized by this co‐precipitation method is 42.91%, the highest among those ever reported for Cs_2_NaInCl_6_:Mn^2+^ crystals (Figure S11, Supporting Information).^[^ ^15^, ^16^ ^]^

To obtain further insights into the orange emission after Mn^2+^ incorporation, Cs_2_NaInCl_6_:Mn^2+^ PCs were PL tested in the 293 to 423 K temperature range. As illustrated in Figure 2d, Cs_2_NaInCl_6_:Mn^2+^ PCs maintain a PL band at any temperature, indicating the stability of the structure. The temperature substantially influences the PL intensity, resulting in a notable reduction from 293 to 523 K (Figure 2e; Figure S12a, Supporting Information). This behavior is similar to other semiconductors, wherein lower temperatures inhibit nonradiative recombination, resulting in higher PL intensity. The decreased PL intensity with the increased temperature would be caused by the thermal quenching effect due to the strengthened electron‐phonon coupling at a higher temperature, especially for double perovskites with a soft lattice.^[^ ^27^ ^]^ As the temperature rises, the PL blue shift is attributed to thermal lattice expansion (Figure 2f). The full width at half maximum (FWHM) increases noticeably, attributable to the strong interaction between excitons and phonons.^[^ ^4^, ^12^ ^]^ Figure 2e presents a temperature‐dependent mapping of PL intensity, wavelength, and FWHM in 3D. In addition, the sample is almost minimally intense at 523 K. After 14 days, the fluorescence intensity returned to 73% of its original value, indicating that the sample has the potential to be recycled. (Figure S12b, Supporting Information).

**Figure 3:**
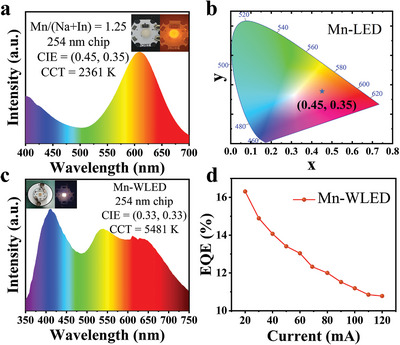
a). The EL spectrum of this LED was fabricated by combining the 254 nm d‐UV chip and Cs_2_NaInCl_6_:Mn^2+^ PCs. b) CIE color coordinates of the LED fabricated by combining the 254 nm UV chip and Cs_2_NaInCl_6_:Mn^2+^ PCs. c) The EL spectrum of the white LED. d) The EQE of the white LED.

Based on the PL spectra (λ_ex_ = 251 nm), the Commission Internationale de L'Eclairage (CIE) values can be calculated to be (0.45, 0.38), (0.50, 0.39), (0.52, 0.40), (0.53,0.39), (0.53, 0.38), and (0.53, 0.37), respectively. (Mn/(Na+In) = 0.25, 0.50, 1.00, 1.25, 1.50, and 2.00), which appeared in the orange‐red area of the CIE (Figure S13a, Supporting Information). The color purity of Cs_2_NaInCl_6_:Mn^2+^ PCs can be calculated from the Equation (3):^[^ ^27^ ^]^

(3) $$Color\;purity\;=\sqrt{\frac{{\left(x-x_{i}\right)}^{2}+{\left(y-y_{i}\right)}^{2}}{{\left(x_{d}-x_{i}\right)}^{2}+(y_{d}-{y_{i)}}^{2}}}$$

where (*x, y*), (*x_i_, y_i_*), and (*x_d_, y_d_*) correspond to the CIE color coordinates of the PCs, white illumination, and dominant wavelength, respectively. The highest color purity was calculated to be 81.46%. The color purity indicated that it could manufacture LEDs for general lighting.^[^ ^31^ ^]^ To further test and verify the application of Cs_2_NaInCl_6_:Mn^2+^ PCs for LEDs, the orange‐red LED was fabricated by combining the 254 nm d‐UV chip and Cs_2_NaInCl_6_:Mn^2+^ PCs. The electroluminescence (EL) spectrum of this LED driven by the current shows a broad peak maximized at 613 nm, consistent with the PL peak profile of the Cs_2_NaInCl_6_:Mn^2+^ PCs (**Figures** **3**a and 2a). The driving current is 150 mA, and the corresponding output power is 1 W. The orange‐red LED produces CIE color coordinates of (0.45, 0.35) with a correlated color temperature (CCT) of 2361 K (Figure 3b). To expand its applications in lighting further, the Cs_2_NaInCl_6_:Mn^2+^ PCs were combined with blue and green commercial phosphors and made into white LEDs using the same method. Figure 3c shows the EL spectrum of this white LED driven by current. The driving current is 20 mA, and the corresponding output power is 0.117 W. The LED produces CIE color coordinates of (0.33, 0.33) with a CCT of 5481 K and a high CRI of 92.3, emitting warm white light (Figure S13b, Supporting Information). Meanwhile, we tested the EQE of Mn‐WLED. The white LED has a maximum EQE of 16.3% (Figure 3d). These results indicate that the LED manufactured by Cs_2_NaInCl_6_:Mn^2+^ PCs has favorable optical performance, which proves that it has a broad application prospect in LED lighting.

**Figure 4:**
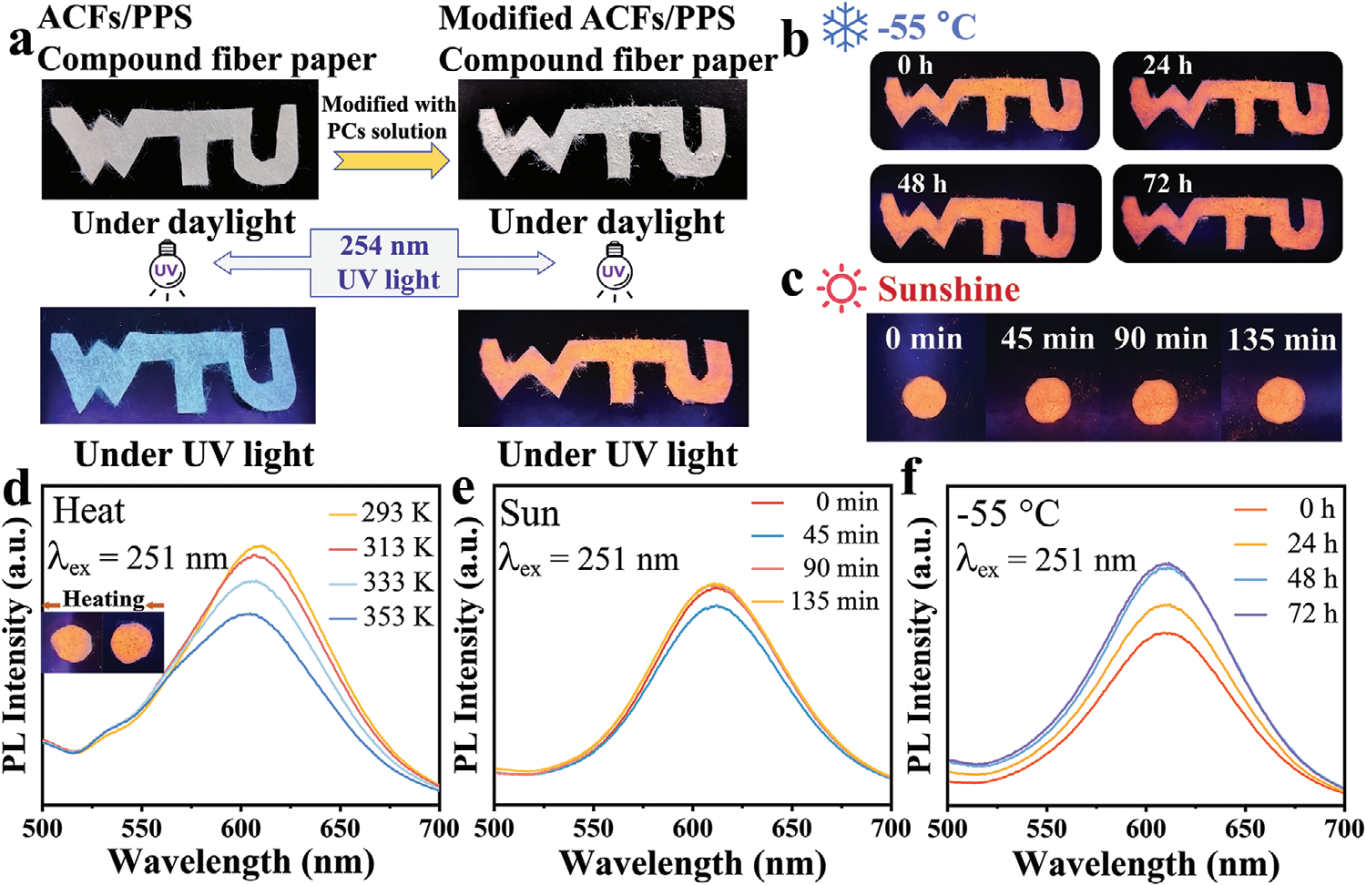
a). Fluorescence properties of ACFs/PPS compound fiber paper without modification and modified with Cs_2_NaInCl_6_:Mn^2+^ PCs. b) Fluorescence properties of composite fiber paper in the −55 °C environment. c) Fluorescence properties of compound paper under sunshine. d) PL spectra of compound papers are in the range of 293 to 353 K. e) PL spectra of compound fiber paper under sunshine. f) PL spectra of compound paper at −55 °C.

Meanwhile, the ACFs/PPS composite fiber paper was modified with Cs_2_NaInCl_6_:Mn^2+^ PCs solution. Based on our previous inquiry, ACFs and PPS compound fiber paper are synthesized via a unique wet paper‐making process (**Figure** **4**a).^[^ ^20^ ^]^ Figure 4b shows that the modified ACFs/PPS compound fiber paper is white under daylight and emits bright orange‐red light under UV light, while the unmodified paper shows dark blue light. The PL spectrum is shown in Figure S14a (Supporting Information). In addition, the composite modified ACFs/PPS composite paper must maintain considerable fluorescent luminescence performance under different environmental conditions to explore its application in wearable flexible luminous materials. When the modified fluorescent fiber paper was placed in the air for 168 h at room temperature, no significant attenuation and quenching of its fluorescence properties were observed (Figure S15, Supporting Information). To further explore the fluorescence behavior of fluorescent fiber paper in more environments, modified ACFs/PPS compound paper was used in progressive heating and continuous low‐temperature testing. During the heating process from 293 to 393 K, the PL intensity of the modified compound paper gradually decreases caused by the thermal quenching effect due to the strengthened electron‐phonon coupling at higher temperatures, which is the same as the performance of Cs_2_NaInCl_6_:Mn^2+^ PCs (Figures 4d and 2d). Although the temperature increase decreased the fiber's fluorescence intensity, it remained more than 75% below 100 °C. The composite fiber was frozen at −55 °C for 48 h; its fluorescence intensity increased significantly and tended to be stable after 48 h (Figure 4b,f). This is due to the low‐temperature inhibition of non‐radiative recombination, which substantially increases the fluorescence intensity of composite fluorescent fiber paper at low temperatures. These results show that the composite fiber maintains good fluorescence properties under high or low‐temperature environments. In addition, the modified fiber paper also shows excellent stability in direct sunlight. The fiber filter paper was exposed to outdoor direct sunlight for 135 min intermittent detection every 45 min. The PL spectra showed that the slight fluctuation in the fluorescence intensity during the process was almost unchanged overall (Figure 4c,e). The fiber paper was placed in direct sunlight continuously for 6 h, and its fluorescence intensity decreased by 5% (Figure S14b, Supporting Information). The result can be attributed to reduced fluorescence intensity as sunlight heats it. Through the above tests, the fluorescent fiber has outstanding fluorescence stability in daily living environments and broad development prospects in flexible wearable function fibers.

**Figure 5:**
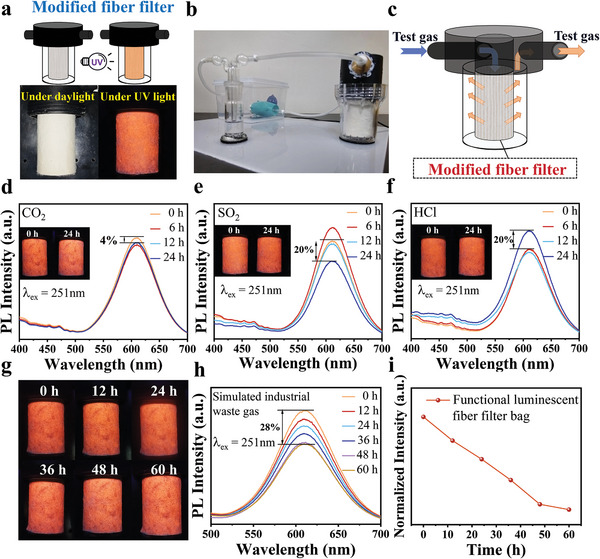
a). The filter and modified ACFs/PPS compound paper filter bag are under daylight and UV lamp. b) Simulated gas circulation equipment. c) Structure diagram and working principle of filter. d) PL spectra of modified ACFs/PPS composite fiber filter bag in CO_2_ circulating gas environment. e) PL spectra of modified ACFs/PPS composite fiber filter bag in SO_2_ circulating gas environment. f) PL spectra of modified ACFs/PPS composite fiber filter bag in HCl circulating gas environment. g) The fluorescence changes of fiber filter bags in simulated industrial waste gas. h) PL spectra of modified ACFs/PPS composite fiber filter bag in simulated industrial waste gas. i) Fluorescence intensity changes of composite fiber paper in simulated industrial waste gas.

High‐performance PPS filter bags often filter fine solid particles in industrial waste gas.^[^ ^33^, ^35^ ^]^ It is difficult to check when the filter bag may be damaged during use because the filter tank is not transparent, and the outer wall cannot be removed at will. The feasible industrial solution is proposed based on the above flexible luminescent fibers. The modified fiber material is made into filter bags and installed on the filter. The filter bag is white under the fluorescent lamp. In contrast, the filter bag emits bright orange and red light, which can accurately judge whether it is damaged by combining the photosensitive resistance of the unique wavelength (**Figure** **5**a). Meanwhile, we independently designed and built a set of simulated industrial waste gas filtration equipment to explore whether the composite fiber fluorescent filter bag can maintain confident fluorescent luminescence performance in the waste gas environment (Figure 5b,c). The device uses a low‐power fan as the circulation part, the filter as the test part, and the liquid that does not react with the circulation gas as the observation part (Figure S16, Supporting Information). The simulated industrial waste gases are prepared with a solution of volatile gases and a simple chemical reaction. In addition, considering that harmful gases may pollute the environment, the entire test set is completely sealed to ensure that toxic gases do not leak into the atmosphere. At the end of the test, the test gas will be recovered and disposed of later. Through this device, to simulate the use environment of filter paper in the industrial acid waste gas, the single industrial waste gas (CO_2_, SO_2_, HCl) cycle test of fiber filter paper was carried out for 24 h. The fiber filter bag was placed in the CO_2_ gas environment for 24 h, and the luminescence of the fiber filter bag was almost no different. No noticeable darkening or quenching was found (Figure 5d inset). The PL spectra similarly showed that the luminescence intensity of the filter bag did not decrease significantly during the test (Figure 5d). There was no fluorescence quenching after the filter paper was circulated by SO_2_ gas (Figure 5e inset). However, the PL spectrum showed that the fluorescence intensity of the circulating SO_2_ gas environment in the fiber filter bag decreased by ≈20% (Figure 5e). This result can be attributed to the fact that the SO_2_ gas destroys the PPS fibers and ruptures them, detaching the perovskite in the compound and decreasing fluorescence intensity.^[^ ^36^ ^]^ Before and after the fiber filter paper is circulated in the hydrogen chloride gas environment for 24 h, the fluorescent light of the modified composite fiber filter bag emits bright orange and red light without fading or quenching under the UV lamp (Figure 5f inset). The PL spectra exhibit that the PL intensity not only did not decrease but was significantly enhanced (Figure 5f). This result can be attributed to hydrogen chloride inhibiting partial hydrolysis of perovskite. The water vapor in the air will hydrolyze the ions in the perovskite, which will destroy its structure and affect its fluorescence intensity, and hydrogen chloride gas can inhibit its hydrolysis and improve the fluorescence luminescence intensity of the filter paper.^[^ ^6^ ^]^ The final filter bag was recycled in the simulated industrial waste gas (CO_2_, SO_2_, HCl) for 60 h, and its fluorescence intensity first decreased and then gradually remained unchanged, with a decrease of ≈28% (Figure 5h,i). Although the PL intensity was reduced, no noticeable quenching and darkening were observed in our fiber filter paper, indicating that it is still valuable to use fluorescence to detect damage (Figure 5g). This fluorescent fiber filter bag expands the application field of perovskite‐modified composite fiber and enhances its use value by combining it with actual chemical production.

## 3 Conclusion

In this study, Cs_2_NaInCl_6_:Mn^2+^ PCs were synthesized by the energy‐efficient co‐precipitation method. Cs_2_NaInCl_6_ PCs doped with Mn^2+^ showed orange‐red emission, with an emission peak wavelength of 613 nm. These crystals have good PL characteristics, with PLQY reaching as high as 42.91% and the average fluorescence lifetime reaching as high as 17.90 ms. Mn‐doped Cs_2_NaInCl_6_ perovskite crystals were prepared using UV 254 nm LED chips to prepare perovskite LEDs. The white LED has a color rendering index of 92 and an EQE of 16.3%. At the same time, perovskite crystals were used to post‐process ACFs/PPS composite fiber paper. The modified ACFs/PPS flexible light‐emitting fiber paper can maintain fluorescence stability at high and low temperatures. A fluorescent filter bag was designed and manufactured based on the fiber composite technology. When the filter bag is illuminated with a specific light, the filter bag is damaged if the detector cannot detect the fluorescence of the local area. At the same time, filter bags can maintain good fluorescence characteristics in some industrial simulation waste gas (CO_2_, SO_2_, HCl) environments for 60 h. The improved method and application provide ideas for the automatic damage detection of filter bags in industrial waste gas filtration and expand the application of perovskite in environmental protection.

## 4 Experimental Section

### Materials

Chemicals used include cesium chloride (CsCl, MACKLIN 99.99%), sodium chloride (NaCl, MACKLIN 99.9%), anhydrous indium chloride (InCl_3_, MACKLIN 99.9%), anhydrous manganese chloride (MnCl_2_, Aladdin 99%), hydrochloric acid (HCl, Sinopharm 37%), polyphenylene sulfide resin n (PPS, Deyang Chemical Co., Ltd.), aramid chopped fibers (ACFs, China BlueStar Chengrand Research Institute of Chemical Industry), sodium metabisulfite (Na_2_S_2_O_5_, Bide Pharmaceuticals 99%), calcium carbonate (CaCO_3_ Sinopharm 99.9%), polyethylene oxide (PEO Sumitomo Keiretsu Co., Ltd.), anionic polyacrylamide (APAM China Pharmaceutical Group Chemical Reagent Co., Ltd.), sodium dodecylbenzene sulfonate (SDS China Pharmaceutical Group Chemical Reagent Co., Ltd.). All chemicals were used without further purification.

### Preparation of Cs_2_NaInCl_6_: Mn^2+^ Perovskite Crystal

The Cs_2_NaInCl_6_:Mn^2+^ PCs with various Mn^2+^‐doping concentrations were synthesized using a co‐precipitation method. In a typical procedure, 2.4 mmol CsCl, 1.2 mmol NaCl, 1.2 mmol InCl_3_, and 3.6 mmol MnCl_2_ and 10 mL of hydrochloride solution (12 mol L^−1^) were loaded into a 50 mL three‐necked flask and heated to 80 °C for 1 h by an oil bath. The original solution was purified and separated by centrifugation (5 min at 6000 rpm). After that, the supernatant was discarded, and the particles were dried in a vacuum furnace at 60 °C for 4 h. The Cs_2_NaInCl_6_:Mn^2+^ PCs with various Mn^2+^‐doped concentrations could be obtained by varying the molar feed ratio of Mn/(In+Na) while maintaining the total molar amount of In^3+^ and Na^+^ unchanged.

### Preparations of Perovskite Crystals LED

Before starting the LED packaging testing, the luminous material and light‐curing glue was must prepared. First, the luminescent material and the glue are evenly mixed in a particular proportion to ensure that the material was fully dispersed in the glue. Then, the bubbles in the mixture were removed by a defoaming machine to ensure the uniformity and stability of the mix. After defoaming the mixture, it was coated onto the LED wafer. In this process, professional glue coating equipment was used to ensure that the glue evenly covers the surface of the wafer. After coating, the wafer was placed in the curing equipment and cured according to the set temperature and time. After the LED packaging was completed, photoelectric test was needed to be conducted. This step was designed to ensure that the luminous performance and stability of the LED meet the requirements.

### Preparation of ACFs/PPS Compound Fiber Paper

First, ACFs and PPS microfiber nonwovens are cleaned with acetone to remove impurities. PPS microfiber pulp was prepared using a grain beater to beat PPS nonwoven fabric. Then, APAM, SDS, and PEO with a mass ratio of 1:3:6 were added to deionized water and stirred at room temperature for 4 h to obtain a uniform dispersion solution (APAMS/SDS/PEO). Then, PPS microfiber pulp and ACFs with a mass ratio of 3:7 were added to the dispersion solution and dispersed by the fiber dissociator. The mixed fiber suspension was formed after uniform distribution and complete dispersion in the dispersion. Finally, the wet papermaking process obtained ACFs/PPS mixed paper with the mixed fiber suspension directly on the paper forming machine.

### Characterization

A PANalytical Empyrean power X‐ray diffractometer with monochromatic Cu Kα radiation measured X‐ray diffraction (XRD) patterns. The scanning electron microscope (SEM) observation with energy‐dispersive X‐ray spectroscopy (EDS) was captured on the Gemini SEM 300, ZEISS, Germany. A trace sample was directly glued to the conductive adhesive, and gold was sprayed 45 s, 10 mA, with Quorum SC7620 sputtering coater. The acceleration voltage was 3 kV for topography and 15 kV for energy spectrum mapping. The detector was an SE2 secondary electronic detector. The electron paramagnetic resonance (EPR) spectrum was carried out using a Bruker EMXplus‐6/1 spectrometer operated at RT with a 9.845 GHz microwave frequency and a power of 0.2 mW. Spectrum was collected at 30 dB with a scan range between 3000 and 3700 G. Photoluminescence spectra were carried out by a Fluorescence spectrophotometer (F‐4700, Hitachi, Japan) in a room with a xenon lamp as the excitation source. PLQY and TRPL decay curves were carried out using a Steady/Transient Fluorescence spectrometer (FLS1000, Edinburgh, England) with a PLQY accessory and a time‐correlated single‐photon counting lifetime spectroscopy system. A Janis VPF‐800 vacuum liquid nitrogen cryostat with a temperature controller was used to mount the Cs_2_NaInCl_6_:Mn^2+^ powder sample for the temperature‐dependent PL measurements.

## Conflict of Interest

The authors declare no conflict of interest.

## Author Contributions

The idea and concept of the current paper were created by X.Z., K.N., and X.M. They also worked on the study design strategy and chose the discussion subjects. The primary experiment and paper writing were finished by X.Z. K.N., X.W., and X.M. provided advice and supervision while editing and revising the final manuscript. After conducting literature searches, Z.H. and X.D. extracted the data from the full‐text papers that met the eligibility criteria. Tests and advice for experiments were given by X.Z. L.M., L.W., and H.W. oversaw the investigation. All the authors discussed the results and commented on the manuscript.

## Supporting information

Supporting Information

## Data Availability

The data that support the findings of this study are available from the corresponding author upon reasonable request.
