# Supplementary material for: High Stability and Corrosion‐Resistant Gas of Recyclable and Versatile Manganese‐Doped Lead‐Free Double Perovskite Crystals toward Novel Functional Fabric and Photoelectric Device
Source: Adv Sci (Weinh). 2024 Jun 14;11(31):2403352. doi: 10.1002/advs.202403352 (PMC11336895; doi:10.1002/advs.202403352)
Supplement: Supplementary file 1 — Supporting Information [file ADVS-11-2403352-s001.docx]

Supporting Information

High Stability and Corrosion-Resistant Gas of Recyclable and Versatile Manganese-Doped Lead-Free Double Perovskite Crystals toward Novel Functional Fabric and Photoelectric Device

*Xiaoman Zhang, Xuyi Wang, Kun Nie*, Xiuqiang Duan, Ziyao Hu, Xiaodong Zhang, Lefu Mei*, Luoxin Wang, Hua Wang, Xiaoxue Ma**

**
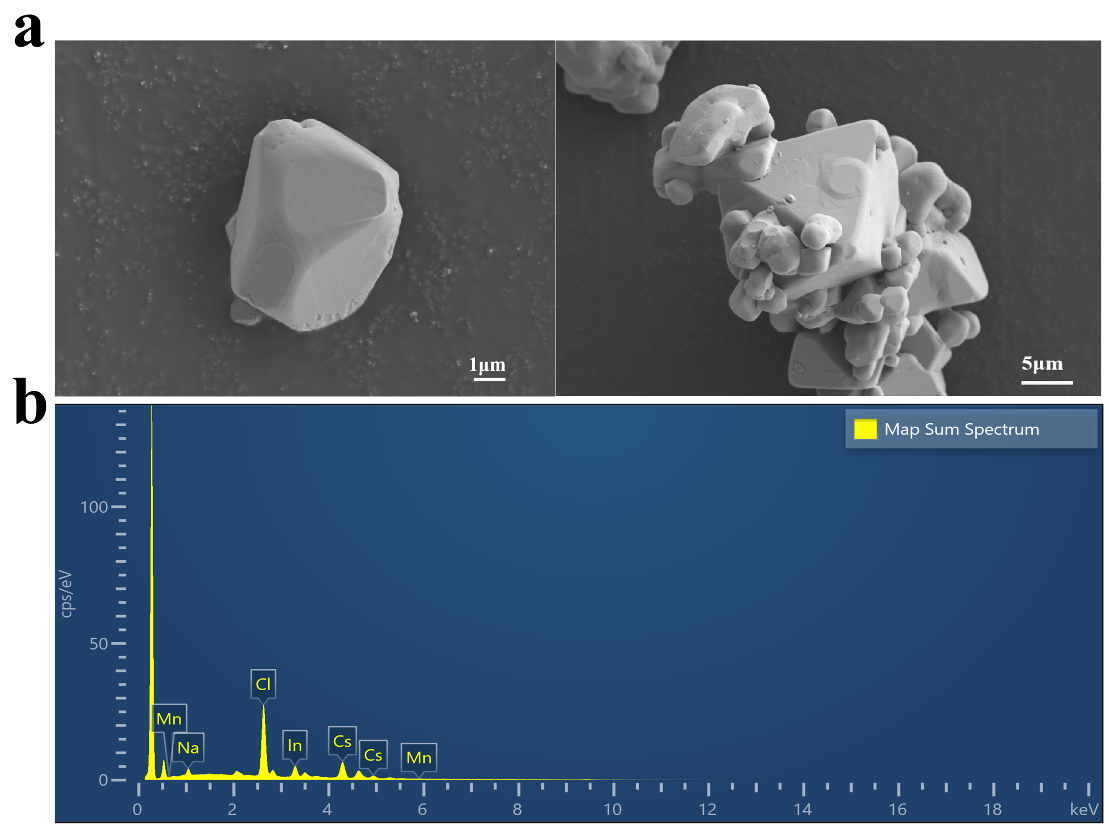
**

**Figure S1.** a) Scanning electron microscope (SEM) image of Cs_2_NaInCl_6_ PCs and SEM image of multiple large crystals in a single crystal agglomeration. b) Map sum spectrum of Cs_2_NaInCl_6_:Mn^2+^.

**
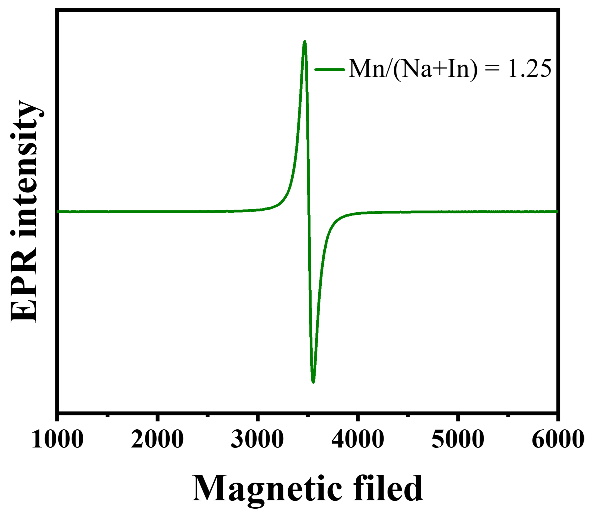
**

**Figure S2.** The room-temperature electron paramagnetic resonance (EPR) spectrum of the Cs_2_NaInCl_6_:Mn^2+^ (Mn/(Na+In) = 1.25).

**
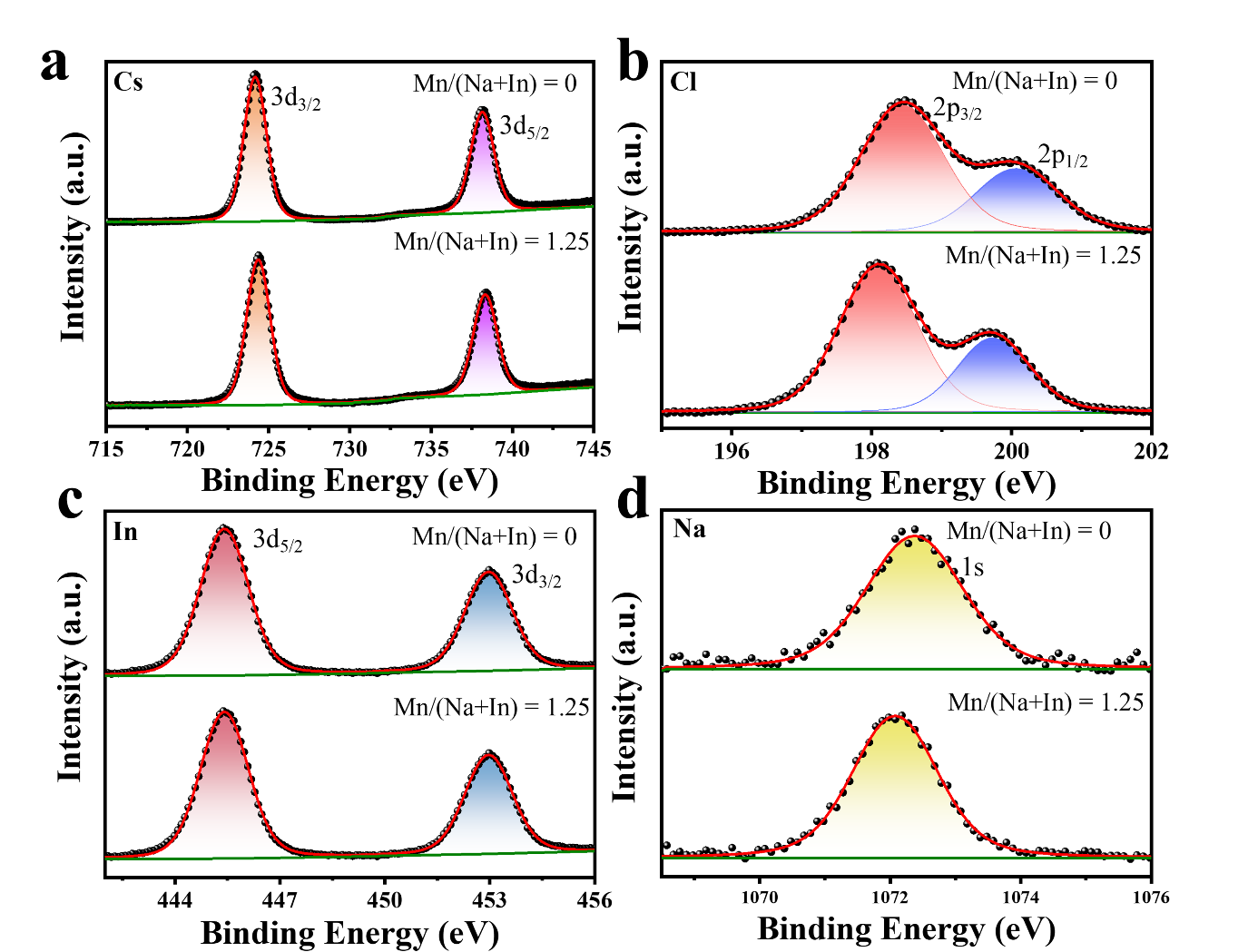
**

**Figure S3.** a) High-resolution X-ray photoelectron spectroscopy (XPS) spectra of Cs. b) High-resolution XPS spectra of Cl. c) High-resolution XPS spectra of In. d) High-resolution XPS spectra of Na.

**
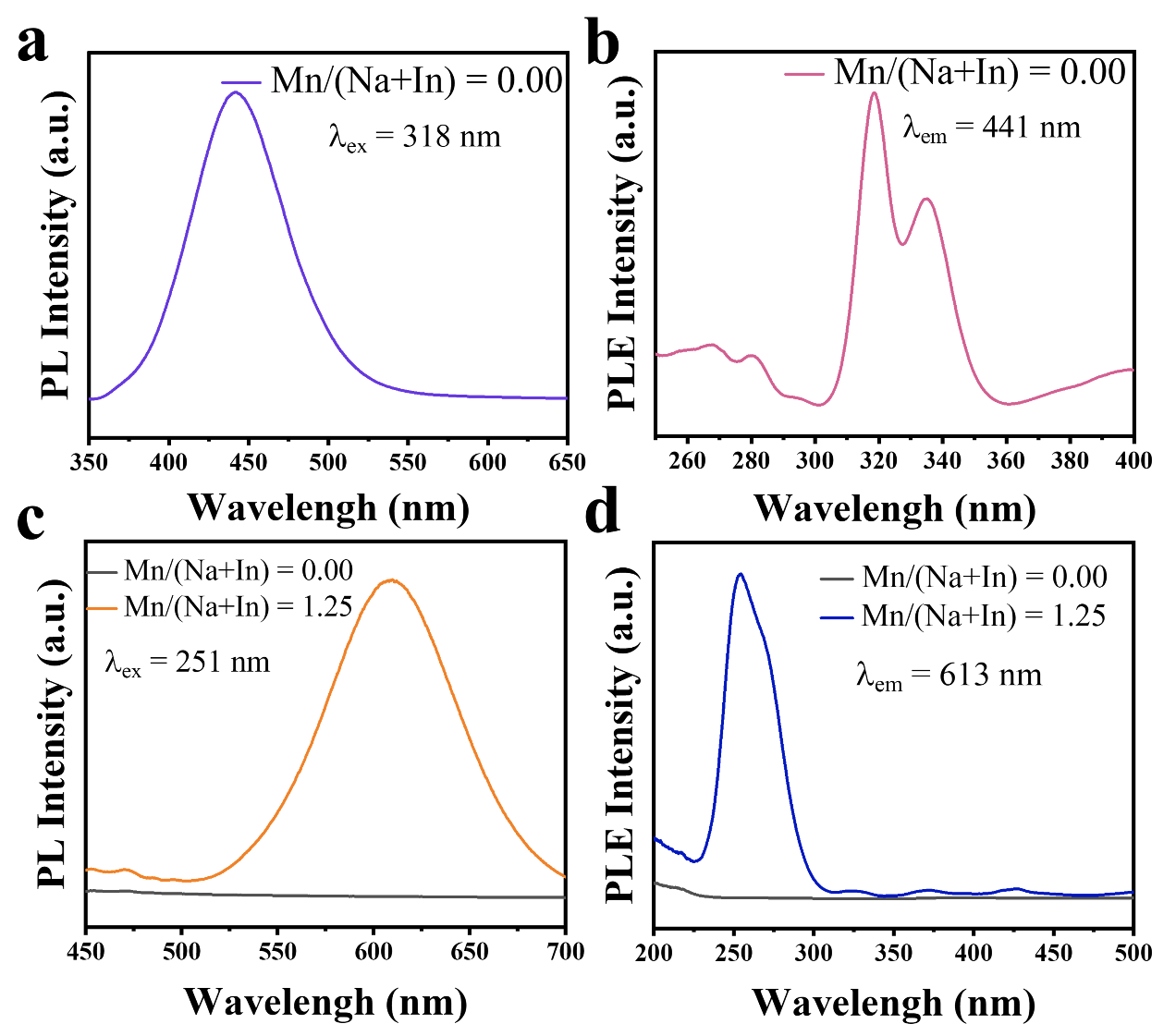
**

**Figure S4.** a) Photoluminescence (PL) spectrum of Cs_2_NaInCl_6_. b) Photoluminescence excitation (PLE) spectrum of Cs_2_NaInCl_6_ single crystal. c) PL spectra of Cs_2_NaInCl_6_ and Cs_2_NaInCl_6_:Mn^2+^ PCs. d) PLE spectra of Cs_2_NaInCl_6_ and Cs_2_NaInCl_6_:Mn^2+^ PCs.

**
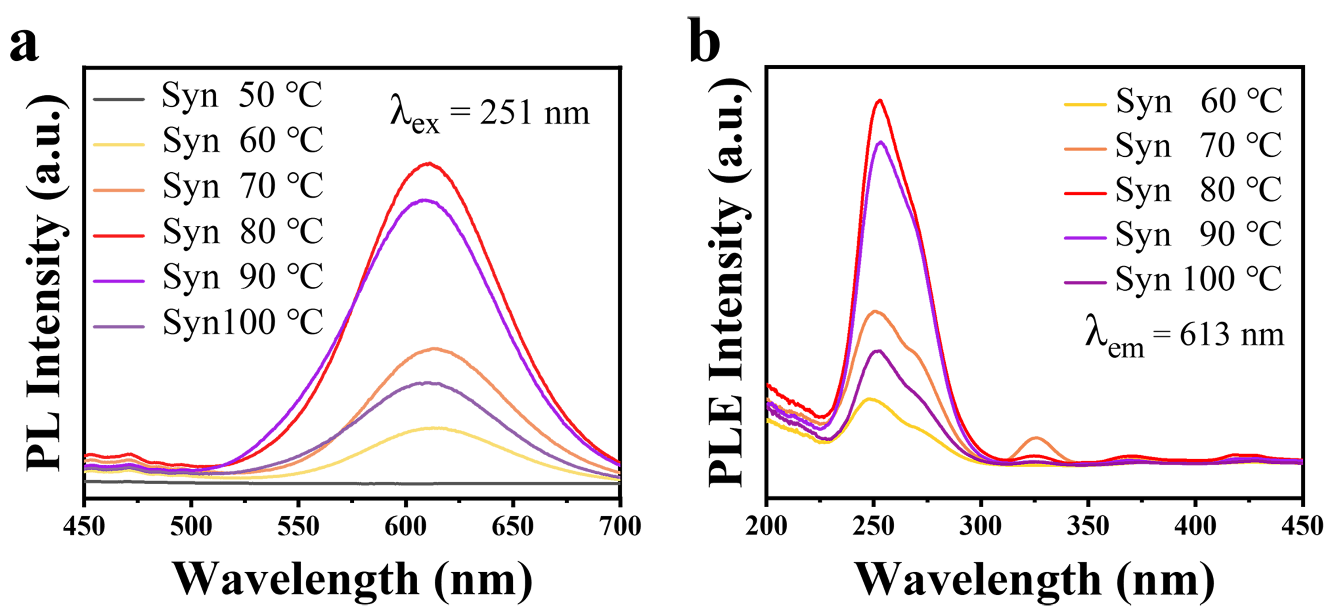
**

**Figure S5.** a) PL spectra of Cs_2_NaInCl_6_:Mn^2+^ PCs synthesized at different temperatures. b) PLE spectra of Cs_2_NaInCl_6_:Mn^2+^ PCs synthesized at different temperatures.

**
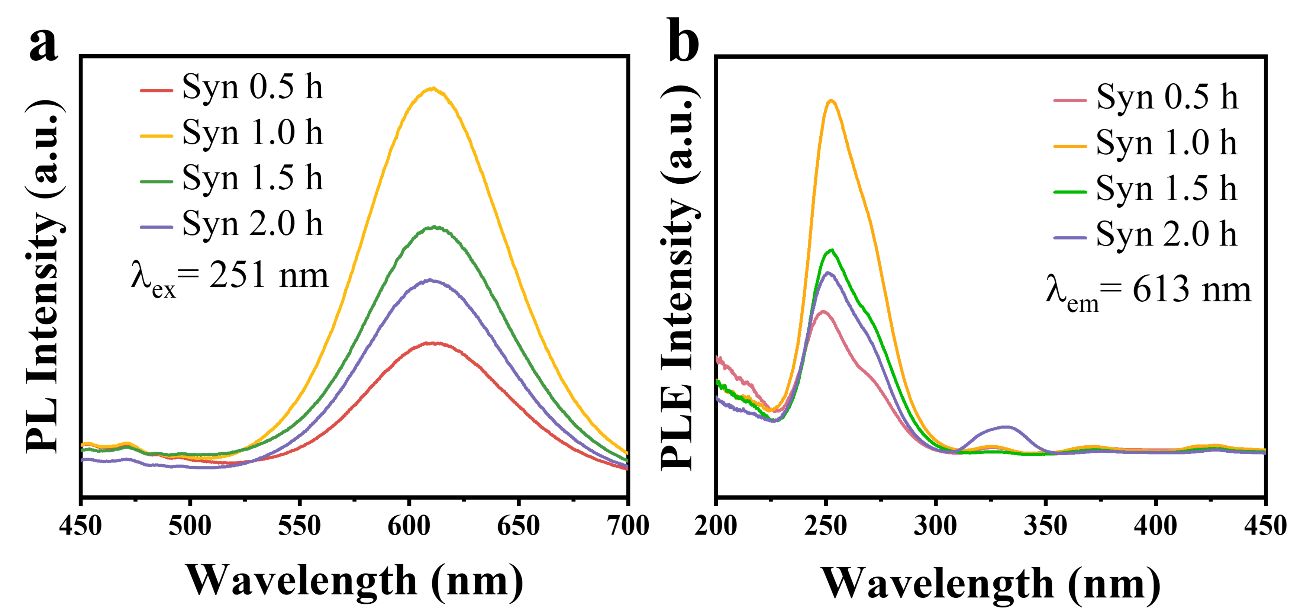
**

**Figure S6.** a) PL spectra of Cs_2_NaInCl_6_:Mn^2+^ PCs synthesized at different times. b) PLE spectra of Cs_2_NaInCl_6_:Mn^2+^ PCs synthesized at different times.

**
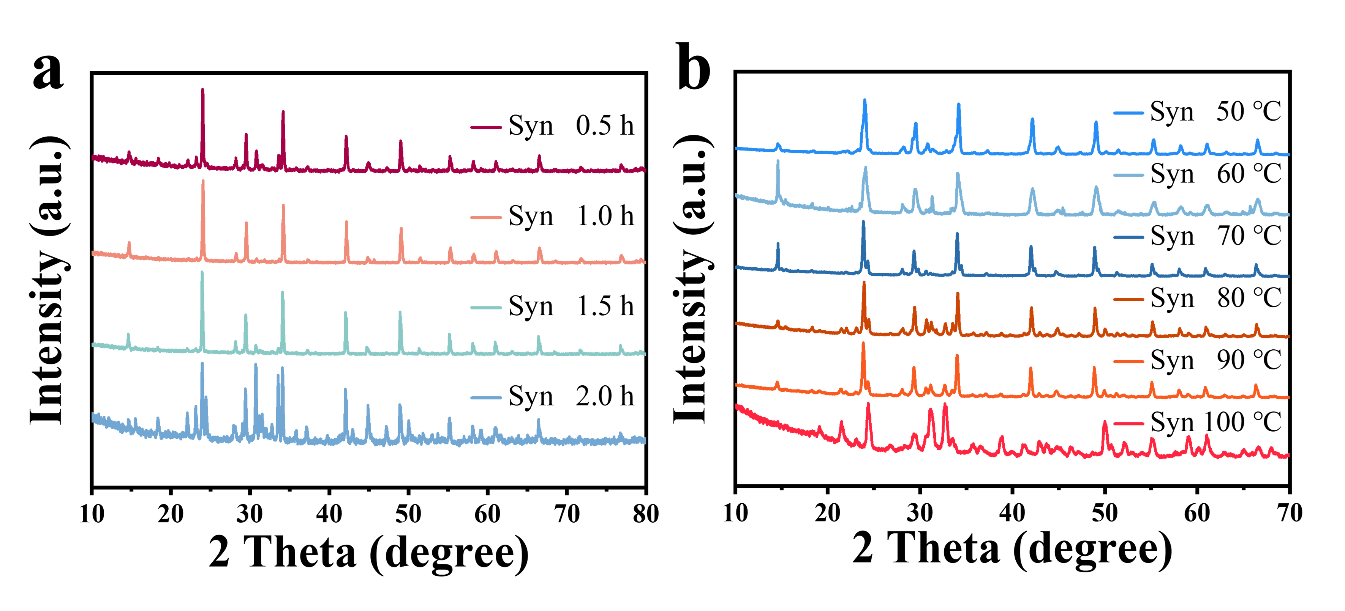
**

**Figure S7.** a) X-ray diffraction (XRD) patterns of Cs_2_NaInCl_6_:Mn^2+^ synthesized at different times. b) XRD of Cs_2_NaInCl_6_:Mn^2+^ synthesized at different temperatures.

**
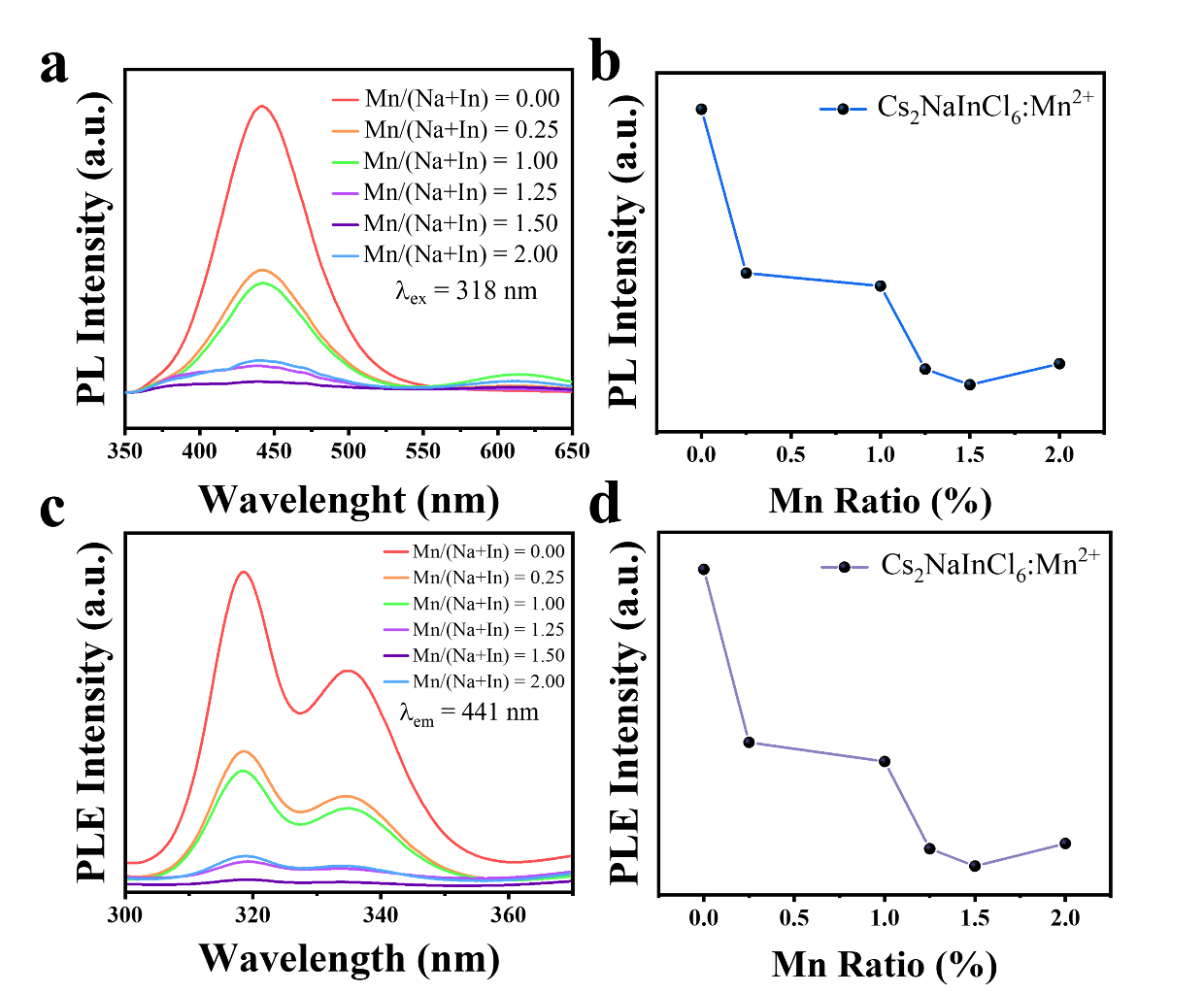
**

**Figure S8.** a) PL (excited by 318 nm UV-light) spectra of Cs_2_NaInCl_6_ and Cs_2_NaInCl_6_:Mn^2+^ PCs. b) Integrated PL intensity as a function of Mn^2+^ ratio. c) PLE (monitored the PL peak at 441 nm) spectra of Cs_2_NaInCl_6_ and Cs_2_NaInCl_6_:Mn^2+^ PCs. d) Integrated PLE intensity as a function of Mn^2+^ ratio.


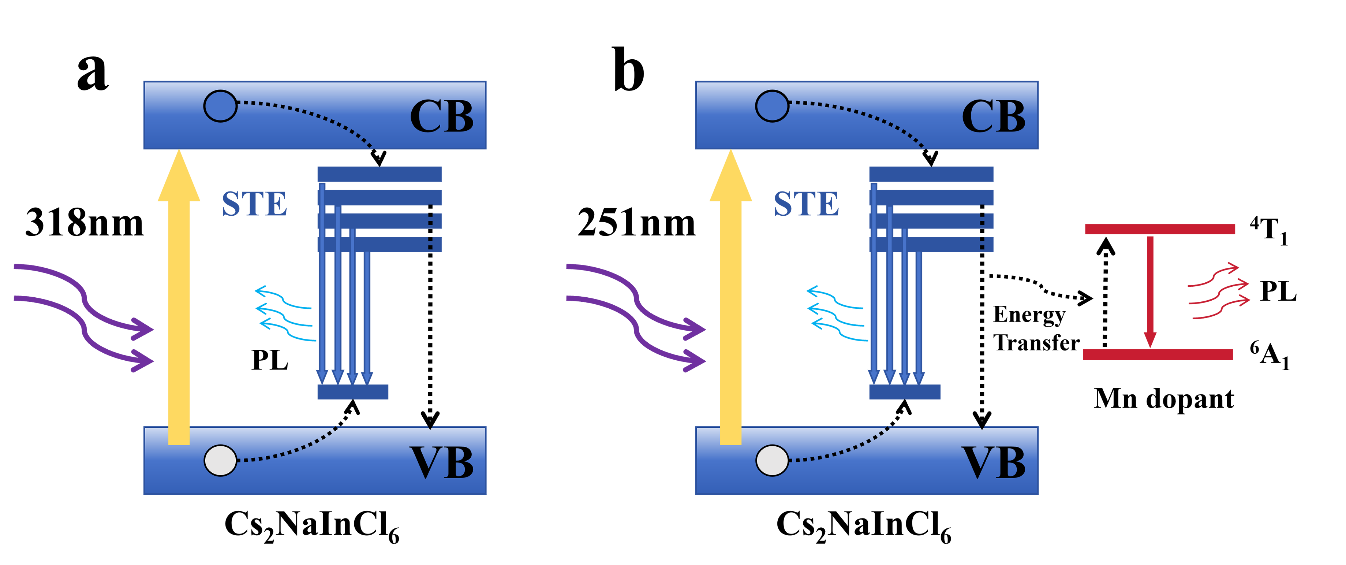


**Figure S9.** a) Energy level diagram of Cs_2_NaInCl_6_. b) Energy level diagram of Cs_2_NaInCl_6_:Mn^2+^.

**
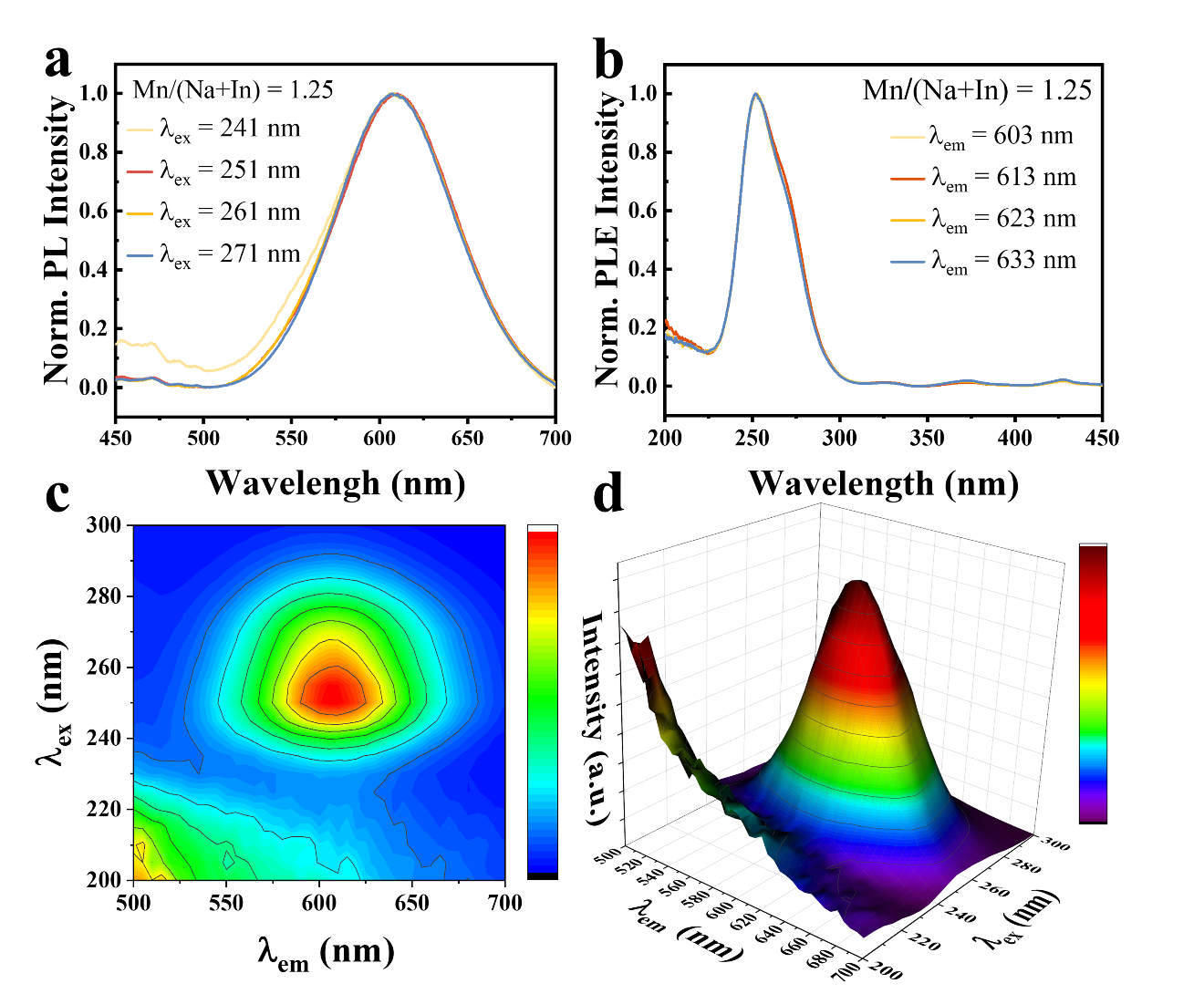
**

**Figure S10.** a) Emission-wavelength-dependent (603 nm-633 nm) PLE spectra of Cs_2_NaInCl_6_:Mn^2+^ PCs. b) Excitation-wavelength-dependent (241 nm-271 nm) PLE spectra of Cs_2_NaInCl_6_:Mn^2+^ PCs. c) Tow-dimensional (2D) fluorescence spectra of Cs_2_NaInCl_6_:Mn^2+^ PCs. d) Three-dimensional (3D) fluorescence spectra of Cs_2_NaInCl_6_:Mn^2+^ PCs.


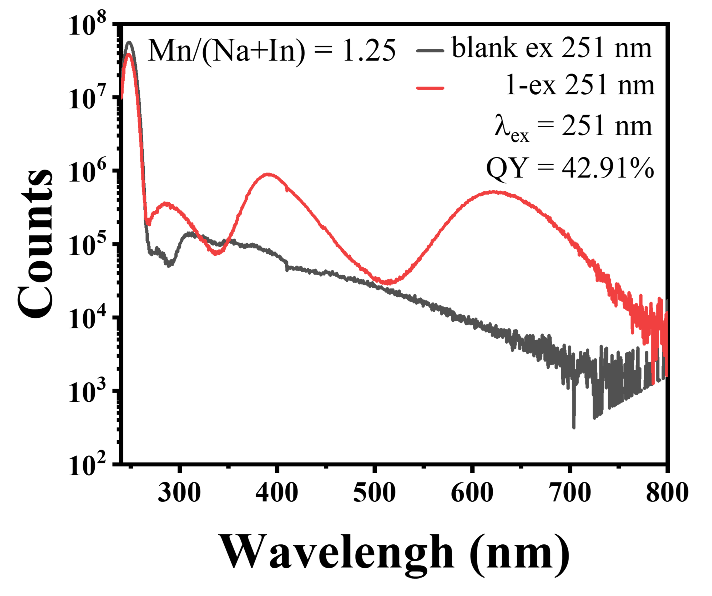


**Figure S11.** Photoluminescence quantum yield (PLQY) of Cs_2_NaInCl_6_:Mn^2+^ (Mn/(Na+In=1.25)) perovskite crystals.

**
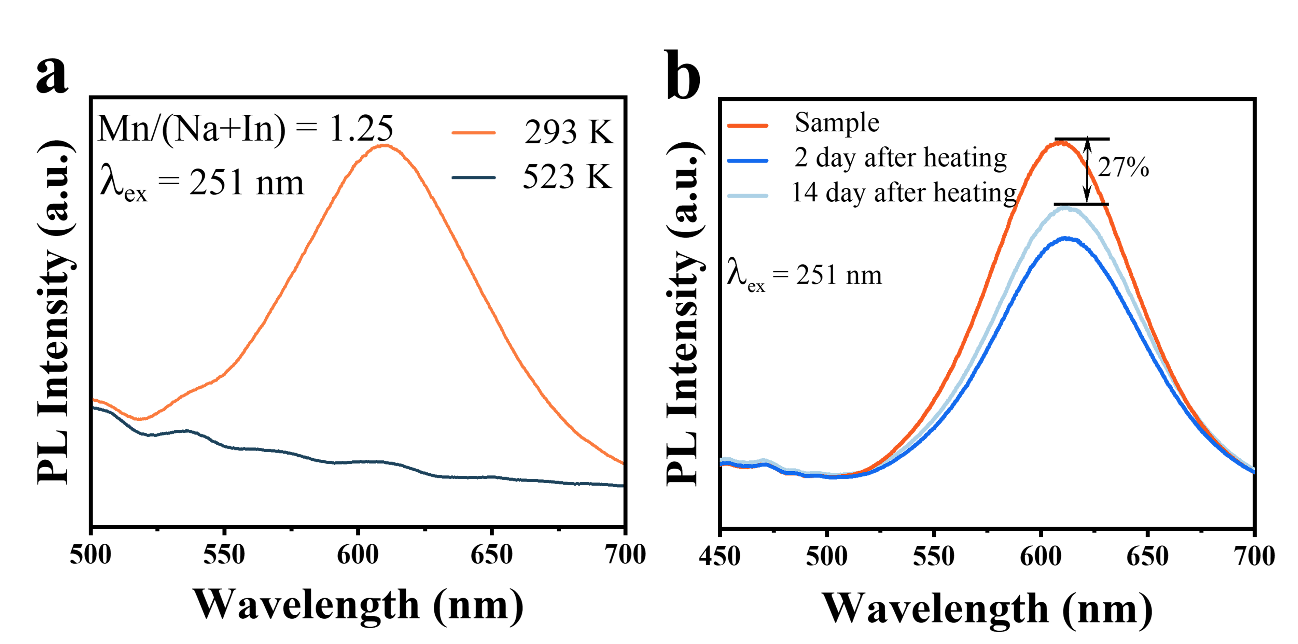
**

**Figure S12.** a) PL spectra of Cs_2_NaInCl_6_:Mn^2+^ PCs at 293 K and 523 K. b) PL spectra of Cs_2_NaInCl_6_:Mn^2+^ PCs after heating test for 2 days and 14 days.


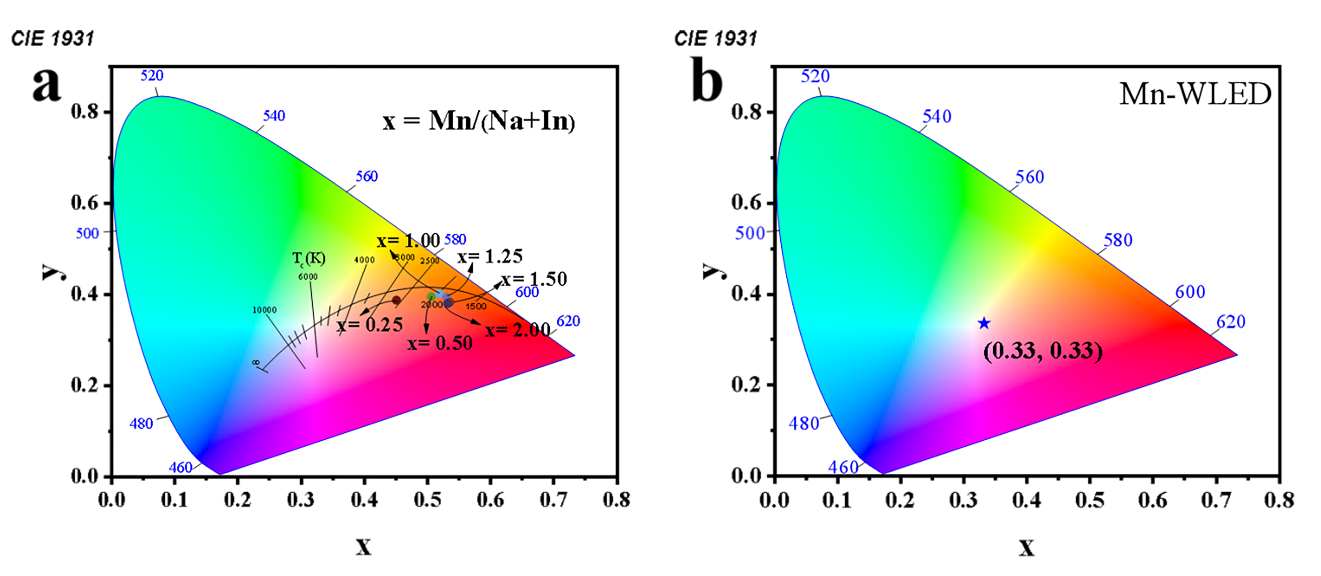


**Figure S13.** a) Commission Internationale de L'Eclairage (CIE) of the Cs_2_NaInCl_6_:Mn^2+^ PCs (Mn/(Na+In) = 0.25, 0.50, 1.00, 1.25, 2.00) PCs. b) CIE of the white LED.


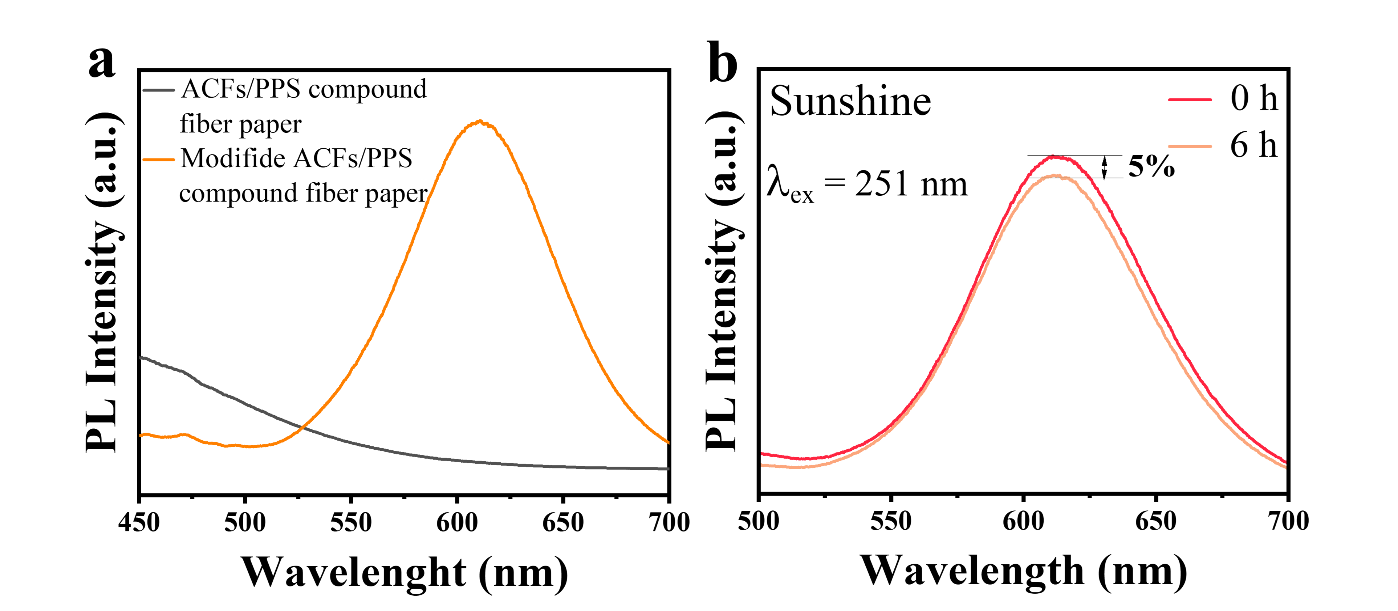


**Figure S14.** a) PL spectra of aramid chopped fibers (ACFs) and polyphenylene sulfide (PPS) compound fiber paper without modification and modified with Cs_2_NaInCl_6_:Mn^2+^ solution. b) PL spectra of compound fiber paper under sunshine for 6 h.


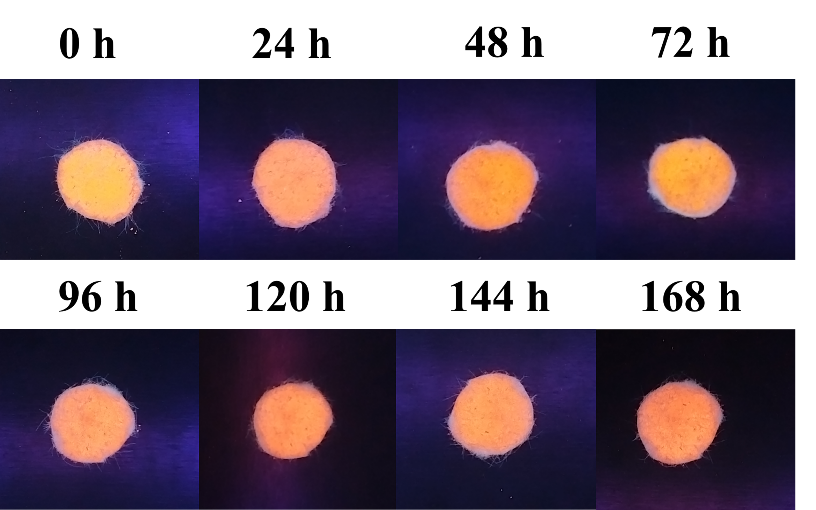


**Figure S15.** Fluorescence performance of modified ACFs/PPS compound fiber paper in air for 168 h.


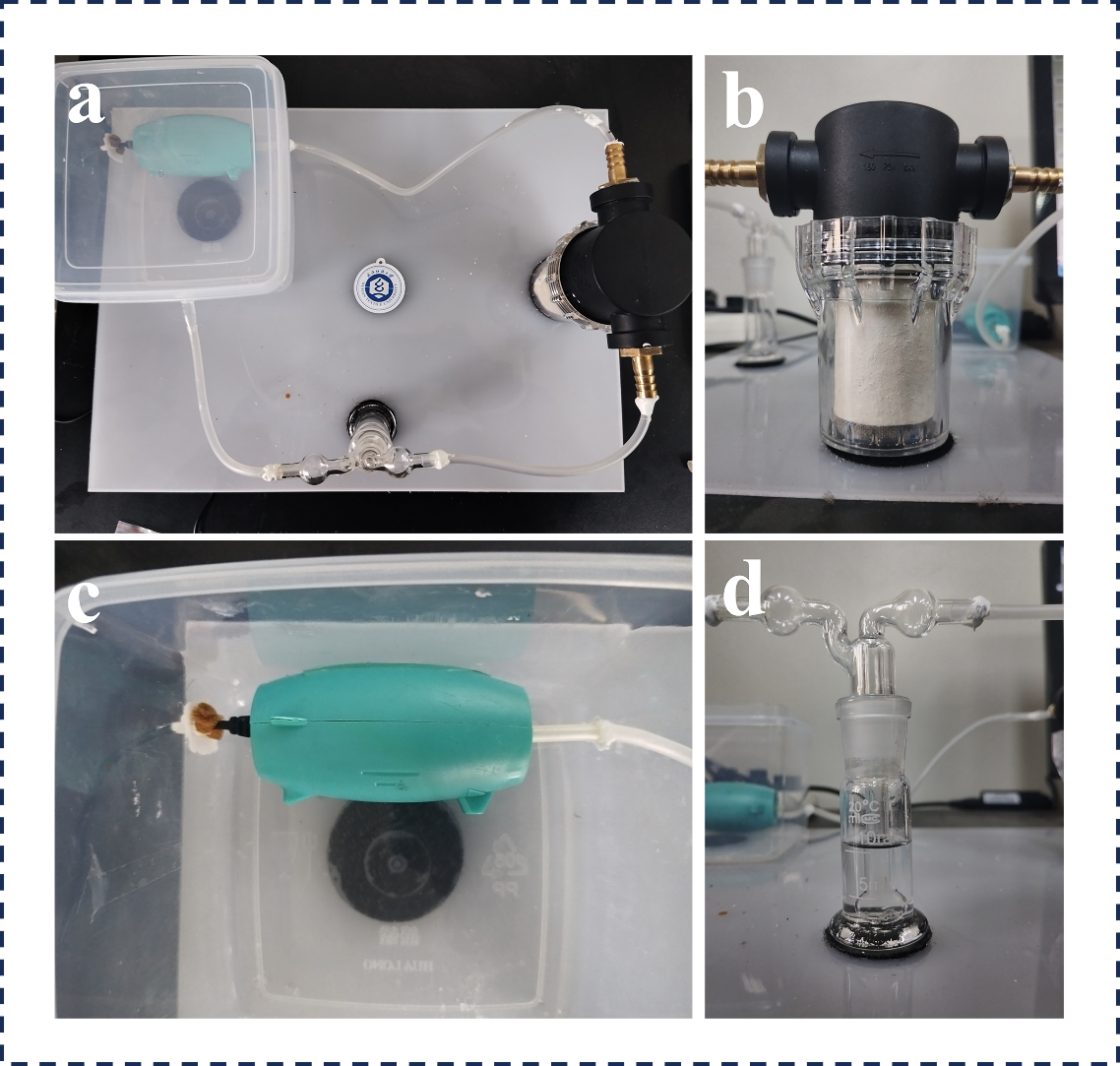


**Figure S16.** a) Planform of the industrial exhaust gas filtration simulator. b) Test part. c) Gas generation part. d) Observation part.
